# Neuroimaging criteria and cognitive performance in vascular mild cognitive impairment: A systematic review

**DOI:** 10.1590/1980-57642015DN94000394

**Published:** 2015

**Authors:** Felipe Kenji Sudo, Gilberto Sousa Alves, Chan Tiel, Letice Ericeira-Valente, Denise Madeira Moreira, Jerson Laks, Eliasz Engelhardt

**Affiliations:** 1 Instituto de Psiquiatria, Universidade Federal do Rio de Janeiro (UFRJ), Rio de Janeiro RJ, Brazil; 2 Departamento de Medicina Clínica, Universidade Federal do Ceará, Fortaleza CE, Brazil; 3 Instituto de Neurologia Deolindo Couto, Setor de Neurologia Cognitiva e do Comportamento-INDC-CDA/IPUB, UFRJ, Rio de Janeiro RJ, Brazil; 4 Serviço de Radiologia, Instituto de Neurologia Deolindo Couto (UFRJ); Hospital Pró-Cardíaco, Rio de Janeiro RJ, Brazil.; 5 Universidade do Estado do Rio de Janeiro, Rio de Janeiro RJ, Brazil.

**Keywords:** cerebrovascular disorders, vascular dementia, cerebral infarction, neurological diagnostic techniques, transtornos cerebrovasculares, demência vascular, infarto cerebral, técnicas de diagnóstico neurológico

## Abstract

The recognition of Cerebrovascular Disease (CVD) at earlier clinical stages may favor the control of vascular risk factors and prevention of dementia. However, operational criteria for symptomatic phases at non-dementia stages are often difficult, as the current criteria normally require the evidence of extensive subcortical disease.

**Objective:**

To identify the neuroimaging profile of Vascular Mild Cognitive Impairment (VaMCI), the impact of those aspects over cognition and the neuropsychological tests that distinguished VaMCI from other groups.

**Methods:**

Searches were performed in Scopus, ISI and PsycINFO, using the following key terms: "vascular mild cognitive impairment" OR "vascular cognitive impairment no dementia" OR "vascular cognitive impairment not demented" OR "subcortical mild cognitive impairment".

**Results:**

Of 249 papers, 20 studies were selected. Ten of those included only patients with severe White Matter Hyperintensities (WMH), whereas 10 others admitted subjects with moderate-to-severe WMH. Both groups showed poor performances in Executive Function (EF) tasks in comparison to normal controls and other diagnostic groups. Among EF tests, those assessing "complex" EF abilities consistently distinguished VaMCI from other groups, regardless of the severity of WMH. VaMCI subjects with severe or moderate-to-severe WMH showed cognitive deficits in comparison with other groups. "Complex" EF tests were the most useful in differentiating those patients from the other groups.

**Conclusion:**

The occurrence of VaMCI may be associated with the presence of CVD at moderate levels; the detection of vascular damage at earlier stages may allow the adoption of therapeutic actions with significant effect-sizes.

## Abstract (pt)

O reconhecimento precoce da Doença Cerebrovascular (DCV) pode permitir o controle de fatores de risco e a prevenção de demência. Contudo, critérios operacionais em seus estágios sintomáticos não-demenciais apresentam problemas, já que critérios atuais requerem a presença de extensa doença isquêmica subcortical.

**Objetivo:**

Identificar o perfil de neuroimagem do Comprometimento Cognitivo Leve Vascular (CCLV), o impacto destes aspectos sobre a cognição e os testes neuropsicológicos que distinguem CCLV de outros grupos.

**Métodos:**

Foram realizadas buscas no Scopus, ISI e PsycINFO, usando a estratégia: "vascular mild cognitive impairment" OR "vascular cognitive impairment no dementia" OR "vascular cognitive impairment not demented" OR "subcortical mild cognitive impairment".

**Resultados:**

De 249 artigos, 20 foram selecionados. 10 destes incluíram apenas pacientes com hiperintensidades de substância branca (HSB) graves, enquanto 10 outros admitiram pacientes com HSB moderadas-a-graves. Ambos os grupos apresentaram desempenho pobre em tarefas de Função Executiva (FE) em comparação com controles normais e outras categorias diagnósticas. Dentre os testes de FE, aqueles que avaliam FE "complexas" diferiram consistentemente CCLV de outros grupos, independentemente da gravidade de HSB. Sujeitos com CCLV e HSB graves ou moderadas-a-graves apresentaram dificuldades cognitivas quando comparados aos demais grupos. Testes que avaliam FE "complexa" foram os mais úteis na diferenciação destes pacientes dos outros grupos.

**Conclusão:**

A ocorrência de VaMCI pode estar associada à presença de HSB moderadas; a detecção precoce do dano vascular permitiria a adoção de medidas terapêuticas com tamanhos de efeito significativos.

## INTRODUCTION

Vascular Cognitive Impairment (VCI) is an umbrella concept which comprises a continuum of vascular-related cognitive impairment, from high-risk preclinical conditions ("brain-at-risk") to Vascular Dementia (VaD). Intermediate stages are commonly referred as Vascular Mild Cognitive Impairment (VaMCI) or Vascular Cognitive Impairment No-Dementia (Va-CIND).^1^ Recent operational criteria, such as the 2011 American Heart Association (AHS)/American Stroke Association (ASA) scientific statement on vascular contributions to cognitive impairment, suggested that the relationship between CVD and cognitive changes could be characterized whether through the evidence of cognitive deficits succeeding a clinical stroke or through identifying vascular lesions on neuroimaging deemed severe enough to explain the cognitive impairment.^2^

More detailed neuroimaging criteria have been described in the 2014 International Society for Vascular Behavioral and Cognitive Disorders (VASCOG) statement for diagnosis of Vascular Cognitive Disorders (VCD). In this document, CVD was evidenced by the presence of one of the following changes:

[1] extensive and confluent subcortical White Matter Hyperintensities (WMH);
[2] large-vessel infarcts: 1 (for Mild VCD) or ≥2 (for Major VCD);
[3] 1 strategically placed infarct (in the thalamus or basal ganglia);
[4] >2 lacunar infarcts outside the brainstem or at least 1 lacune combined with extensive WMH; and
(5) intracerebral hemorrhages: ≥2 or 1 strategically placed.^3^

The VASCOG statement represented a more comprehensive neuroimaging criterion in comparison to the AHA/ASA recommendations and a substantial change in relation to the Erkinjuntti's neuroimaging criteria for Subcortical Ischemic VaD (2000), in which extensive and confluent WMH or moderate WMH combined with at least 5 lacunes was required to characterize CVD.^4^ Nonetheless, the persistence in the new criteria of the need for extensive and confluent WMH contrasted with some studies, which have suggested that moderate WMH with less than 5 lacunes could account for cognitive impairments.^5^ As indicated by several studies, mild WMH is highly prevalent among normal elderly individuals and has not been significantly associated with cognitive changes.^6^

One possible advantage in identifying CVD in its mildest clinical (VaMCI) and neuroimaging (moderate subcortical WMH and less than 5 lacunes) stages is the fact that progression of vascular damage might be preventable. Early detection might allow the adoption of disease-modifying therapies that could prevent the progression of vascular lesions; therefore, it might interrupt the advance of cognitive impairment that could result in VaD. Finally, recent diagnostic criteria for Va-CIND overlap with the ASA/AHA criteria for VaMCI,^7^ thus the term VaMCI has been used in this review to refer to both constructs.

According to the above pondering, a systematic review was undertaken aiming:

[1] to assess the neuroimaging profile of individuals classified as VaMCI in clinical studies;
[2] to determine whether different neuroimaging criteria impact over cognitive findings, and
[3] to identify neuropsychological tests that could distinguish VaMCI from normal controls or other diagnostic groups across studies using different criteria for CVD. The authors hypothesized that the choice of establishing the threshold of brain vascular lesions into moderate or severe stages of WMH may account for divergent cognitive findings among studies.

## METHODS

**Data search and selection.** Studies were found through searches in Scopus, ISI Web Of Knowledge and PsycINFO, using the following key terms, in all fields and published in any date: "vascular mild cognitive impairment" OR "vascular cognitive impairment no dementia" OR "vascular cognitive impairment not demented" OR "subcortical mild cognitive impairment". This search strategy was augmented with hand searches of reference lists of included studies. More articles were obtained from directly contacting authors for relevant papers.

After the searches were performed, articles were included if they were: clinical studies, which included neuroimaging data from individuals with VaMCI; that compared cognitive performances between VaMCI and other diagnostic groups [VaD, AD, non-vascular MCI (non-VaMCI)] or normal controls; and written in English, French, Spanish or Portuguese.

The authors have excluded studies that: classified individuals as VaMCI based solely on clinical/ neuropsychological aspects (e.g., studies in which the cognitive deficits were judged to have vascular cause through clinical features, such as stepwise progression, sudden onset, gait disturbances, focal neurological signs or those that applied only an ischemic score to identify the presence of cerebrovascular disease); did not assess subjects with MCI, defined as those presenting cognitive impairments that do not fulfill criteria for dementia; did not acknowledge a detailed neuroimaging criterion for the diagnosis of VaMCI (e.g., cognitive impairment considered associated with vascular lesions through subjective evaluation from an expert); did not compare cognitive performances between VaMCI and controls or other diagnostic groups; or included subjects with cortical infarction or cortical atrophy suggestive of large-vessel or neurodegenerative diseases. The current study followed the standard protocols of PRISMA statement.^8^

**Data extraction.** Data were extracted from full-texts by one author (FKS) and reviewed by a second author (EE). Divergences were furtherly discussed among the entire team of authors.

## RESULTS

**Figure 1:**
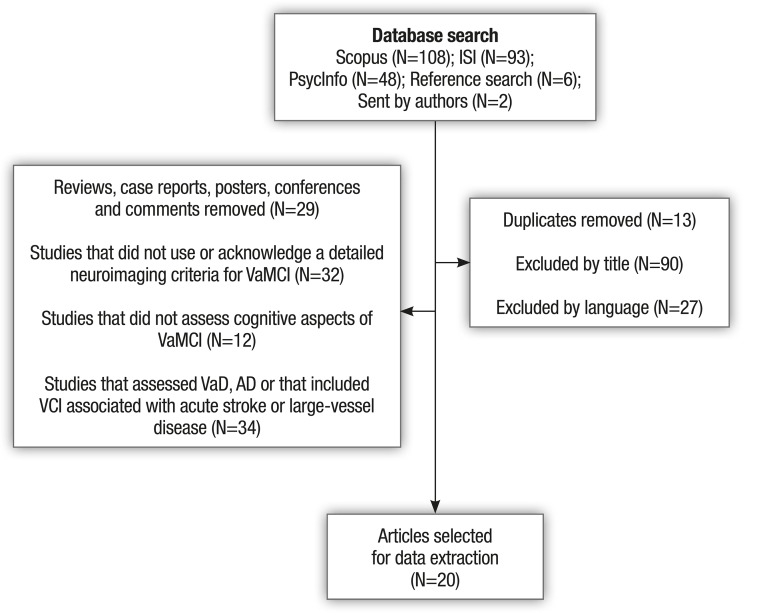
Flow-chart describing the process of study selection.

Of a total of 249 retrieved papers, 20 studies were selected for data extraction. Figure 1 summarizes the stages of data search and selection.

**Clinical criteria for MCI.** Participants in the studies presented objective cognitive deficits and preserved functional status. Mild differences included articles that identified those with cognitive impairments based on performances in screening tests for cognitive deficits (e.g., MMSE ≥ 24, CDR= 0.5, Clock Drawing Test scores lower than 2/6).^9-14^ Cognitive impairment was defined as performances 1 to 2 standards deviations (between the 16^th^ and the 2^nd^ percentile) below mean normative values, in some studies.^15-21^ Few studies, all of them prior to 2009, required impairment in memory for diagnosis of MCI;^17,22,23^ however, most papers did not include any specific cognitive domain or proposed dysexecutive symptoms as typically associated with VaMCI.

**Neuroimaging criteria for subcortical vascular disease.** Ten of the studies classified subcortical CVD as the presence of white-matter changes compatible with severe WMH and/or at least 5 subcortical lacunes. Five of those followed the criteria proposed by Erkinjuntti et al. (2000) for Binswanger's Disease, which requires the presence of severe WMH, periventricular lesions larger than 10 mm and deep WMH equal or over 25 mm of diameter.^11,15,16,24,25^ A modified version of the Computerized Tomography (CT) criterion for Subcortical Vascular Dementia proposed by Erkinjuntti et al. (2000) was applied in two of the studies. CVD, in those cases, was represented by patchy or diffuse leukoaraiosis and at least one lacunar infarct on neuroimaging.^9,22^ Evidence of extensive WMH, defined as lesions larger than 3 mm of diameter in the semioval center and larger than 5 mm in the deep gray nuclei, was the criterion used in one study.^26^ Other methods for identification of individuals with severe WMH included semiautomatic white-matter volumetry techniques. Nordahl et al. (2005) classified individuals with WMH extending for more than 19.375% of total white-matter volume as presenting severe WMH.^23^ Moretti et al. (2008) computed the presence of CVD by counting voxels corresponding to WMH and identifying those individuals whose lesions corresponded to values over the fourth quartile of volume damage.^10^ Table 1 illustrates those findings.

**Table 1:** Studies that included severe (largely confluent) WMH and/or at least 5 lacunes for diagnosis of SVD.

Author, year	N	Groups	Clinical criteria for MCI	Neuroimaging criteria for SVD	Neuropsychological tests
Frisoni et al., 2002	64	VaMCI, VaD, non-VaMCI	Dysexecutive syndrome + memory impairment + unimpaired complex ADL	Patchy WMH or diffuse symmetrical WMH + 1 lacunar infact	WCST, Category fluency, Letter fluency, Token test, Corsi test, Digit span, Prose recall
Galluzzi et al., 2005	43	VaMCI, non-VaMCI	MMSE ≥ 24, CDR=0.5	Patchy WMH or diffuse symmetrical WMH + 1 lacunar infact	WCST, Category fluency, Letter fluency, Corsi test, Digit Span, Prose recall
Nordahl et al., 2005	42	NC, VaMCI, non-VaMCI	Memory complaints, poor perfomances in Memory tasks, preserved global cognitive performances, unimpaired ADL	WMH extension above the 75th percentile (WMH above 19.375% of total white matter volume)	MMSE, Wechsler Memory Scale Revised, Memory Assessment Scales List Learning, BNT, Block design, Digit Span, Category fluency
Shim et al., 2008	57	NC, VaMCI, non-VaMCI	Objectively measured cognitive decline + unimpaired ADL	Severe WMH, periventricular WMH > 10mm, deep WMH ≥ 25 mm	MMSE, 12-word list from HVLT, Digit span, Rey-Osterrieth Complex Figure Test, BNT, Stroop, Category fluency, Letter Fluency, Go-No Go, Luria Loop test
Moretti et al., 2008	116	VaMCI, atrophic MCI, “cholinergic” MCI	Cognitive complaints + MMSE between 24 and 27, or (MMSE of 28 or higher + Clock Drawing Test of 2/6 or worse) + unimpaired ADL	Number of voxels corresponding to WMH above the upper quartile	Rey word list immediate and delayed recall, Trail Making Test A, B and B-A, Clock Drawing Test, Raven matrices, Inverted motor learning, Rey-Osterrieth Complex Figure Test, Category fluency, Letter Fluency, Token test
Fernández et al., 2011	53	NC, VaMCI, non-VaMCI	Petersen (2001), Frisoni (2002)	Extensive WMH (WMH>3 in semiovale nuclei or >5 mm in deep grey nuclei) or diffuse symmetrical WMH + 1 lacunar infact	MMSE, CERAD (Category fluency, BNT, Word list memory test, constructional praxis, TMT A and B), Digit Span, Abstraction, Letter fluency
Bella et al., 2011	20	NC, VaMCI	Not demented (DSM-IV), MMSE ≥ 24	Severe WMH, periventricular WMH > 10 mm, deep WMH ≥ 25 mm	MMSE, Stroop
Kim et al., 2012	48	VaMCI, VaD	Subjective cognitive complaints; objective cognitive decline below the 1 SD on neuropsychological tests; normal general cognitive function; normal ADL; not demented	Severe WMH, periventricular WMH > 10 mm, deep WMH ≥ 25 mm	MMSE, Digit span, Rey-Osterrieth Complex Figure Test, Seoul Verbal Learning Test, Controlled Oral Word Association Test, Stroop
Lee et al., 2014	207	VaMCI, non-VaMCI	Subjective cognitive complaints, normal ADL, cognitive performance < 16th percentile on tests, absence of dementia, focal neurological symptoms/signs	Severe WMH, periventricular WMH > 10 mm, deep WMH ≥ 25 mm	MMSE, Digit span, Rey-Osterrieth Complex Figure Test, Seoul Verbal Learning Test, Controlled Oral Word Association Test, Stroop
Sheorajpanday et al., 2014	57	VaMCI, non-VaMCI	First presentation of cognitive decline, age ≥ 55 years, intact ADL, not VaD (NINDS-AIREN), presumed vascular cause	Severe WMH, periventricular WMH > 10 mm, deep WMH ≥ 25 mm	MMSE, Wechsler Memory Scale III, Wechsler Adult Intelligence Scale III, TMT A and B, Rey-Osterrieth Complex Figure Test, Digit spam, Category fluency, Letter fluency, Raven matrices

Moderate WMH and/or less than 5 lacunes were deemed sufficient to characterize CVD in ten of the studies. Overall, individuals that scored 2 or more in the modified-Fazekas Scale, corresponding to the presence of moderate periventricular WMH ("smooth halo") with beginning confluent deep WMH, were selected for those studies. Identification of at least 2 lacunar infarcts was an alternative criterion for diagnosis of moderately severe cerebrovascular disease. Table 2 depicts those results.

**Table 2:** Studies that included moderate (beginning confluent; smooth halo) WMH and/or less than 5 lacunes required for diagnosis of SVD.

Author, year	N	Groups	Clinical criteria for MCI	Neuroimaging criteria for SVD	Neuropsychological tests
Norlund et al., 2007	180	NC, VaMCI, non-VaMCI	Subjective and objective cognitive impairment for more than 6 months not demented.	Moderate WMH or 2 or more lacunes	Visual Object and Space Perception, Assessment of Subtle Language Deficits, Parallel Serial Mental Operations, TMT
Gainotti et al., 2008	142	NC, VaMCI, non-VaMCI	Long-term Memory performance < 2 scores from cutoff, no cognitive impairment in non-memory domains, preserved functional status	2 or more subcortical infarcts (below 2 cm of size) or 1 subcortical infact + periventricularWMH of any size	RAVLT, Rey-Osterreith Complex Figure, Digit and Spatial Span, phonological and categorical verbal fluency, Raven's Standard Progressive Matrices, Multiple Features Targets Cancellation, Vill's test for temporal rule induction, Stroop interference test
Zhou et al., 2009a	156	NC, VaMCI, non-VaMCI	Cognitive impairment + CDR=0.5 + unimpaired ADL	Wahlund scale ≥ 2 or more than 2 lacunes	MMSE, Digit Span Backwards and Forward, WHO-UCLA AVLT, Rey-Osterreith Complex Figure, Stroop, Semantic Verbal Fluency, WAIS-RC, California Card Sorting Test, CDT
Zhou et al., 2009b	160	NC, VaMCI	Cognitive impairment + CDR=0.5 + unimpaired ADL	Wahlund scale ≥ 2 or more than 2 lacunes	MMSE, Digit Span Backwards and Forward, WHO-UCLA AVLT, Rey-Osterreith Complex Figure, Stroop, Semantic Verbal Fluency, WAIS-RC, California Card Sorting Test, CDT
Norlund et al., 2011	216	VaMCI, non-VaMCI, VaMCI and non-VaMCI with and without biological markers	Cognitive complaint+ objective cognitive decline + not demented + unimpaired ADL	Moderate WMH or 2 or more lacunes	Digit Symbol, TMT, Digit Span, RAVLT, Wechsler's Logical Memory, Rey-Osterreith Complex Figure, Visual Object and Space Perception, Block Design, Token Test, Boston Naming, Semantic Verbal Fluency, Parallel Serial Mental Operations, Dual Task, Stroop, Wisconsin Card Sorting Test, Cognitive Estimation Test
Marra et al., 2011	135	VaMCI, non-VaMCI	subjective and objective cognitive deficits (worse than 1.67 SD from normal values) and normal functional status	Fazekas ≥ 2 or more than 3 lacunes; or Periventricular WMH grade 1 + 2 or more lacunes	Rey's Auditory Verbal Learning Task, Rey-Osterrieth complex figure, Stroop, Multiple Features target cancellation, Phonological and Semantic Verbal Fluency, Raven's Progressive Matrices
Villeneuve et al., 2011	72	NC, MCI with WMH and MCI without WMH	Subjective cognitive complaint + cognitive performance 1.5 SD below normative values + preserved ADL	Wahlund ≥ 2	Mémoria computerized battery, BEM-144, RL/RI word recall Task, Rey-Osterrieth complex figure, Stroop, WAIS-III, Boston Naming Test, Benton judgment of line orientation test
Yi et al., 2012	54	NC, VaMCI	Subjective cognitive complaints, objective cognitive impairments, not demented (DSM-IV), normal ou near-normal functionbal status, CDR= 0.5, MMSE≥ 24.	Moderate to severe WMH in at least 1 region with a Wahlund rating scale score ≥2 and/or multiple pericentricular and deep lacunes	MMSE, AVLT
Sudo et al., 2013	36	NC, VaMCI	Impairment of 1.5 SD below the mean on 1 or more cognitive tests in relation to normative values, preserved or mildly impaired functional activities, (FAQ <5)	Moderate or severe degree of WMH on Fazekas scale and hippocampal atrophy ≤1 on de Leon score (none or questionable atrophy)	MMSE, CAMCOG, CDT, TMT, Semantic Verbal Fluency, Boston Naming Test
Brookes et al., 2015	503	VaMCI, CVD without cognitive impairment	scoring ≤1.5 SD of the normal mean on a given test	lacunar infarcts or lacunar infarcts with leukoaraiosis (Fazekas≥2)	Brief Memory and Executive Test (BMET), MMSE, MoCA

**Cognitive performances and neuroimaging criteria.** Although the choice of neuropsychological tests varied across studies, cognitive assessment in most cases included tasks that measured executive function (EF), memory, language and visuospatial/ visuoconstructive abilities. Table 3 summarizes the main affected cognitive abilities in the selected studies. EF has been divided into 3 components, following studies that performed a latent variable approach of multiple EF measures: "shifting" (switching between tasks), "inhibition" (deliberate overriding of prepotent responses) and "working memory/updating" (monitoring and rapidly changing new contents).^27^ Tests categorized as "less specific EF tests" included tasks that assessed multiple EF dimensions (e.g., Clock Drawing Test, Verbal Fluency etc.), instead of measuring one single aspect of it.^28^ Matching between neuropsychological tests and cognitive domains was made in accordance with evidences in the literature.^21,28-40^ Table 4 summarizes the correspondence between cognitive domains and neuropsychological tests used in the studies.

**Table 3:** Summary of cognitive findings in the selected studies according with the neuroimaging criteria for CVD.

Criteria for CVD	Articles	Affected cognitive functions in studies							
		Shifting	Inhibition	Working memory/ updating	Less specific EF tasks	Visuospatial / Visuoconstructive abilities	Memory	Language	Global cognition
Severe WMH and/or or ≥ 5 lacunes	Frisoni et al., 2002	VaMCI≠VaD**	Non-VaMCI≠VaMCI* VaMCI≠VaD**	n.s.	Non-VaMCI≠VaMCI*	-	n.s.	n.s.	VaMCI≠VaD**
	Galluzzi et al., 2005	n.s.	Non-VaMCI≠VaMCI*	n.s.	Non-VaMCI≠VaMCI*	-	n.s.	-	n.s.
	Nordahl et al., 2005	-	-	n.s.	NC≠VaMCI**	NC≠VaMCI** Non-VaMCI≠VaMCI**	NC≠VaMCI** NC≠non-VaMCI**	n.s.	NC≠VaMCI** NC≠non-VaMCI**
	Shim et al., 2008	-	n.s.	n.s.	Non-VaMCI≠VaMCI*	Non-VaMCI≠VaMCI**	Non-VaMCI≠VaMCI*	Non-VaMCI≠VaMCI*	n.s.
	Moretti et al., 2008	Non-VaMCI≠VaMCI*	-	-	n.s.	Non-VaMCI≠VaMCI*	Non-VaMCI≠VaMCI*	n.s.	-
	Fernández et al., 2011	NC≠VaMCI*	-	n.s.	NC≠VaMCI* NC≠non-VaMCI*	n.s.	NC≠VaMCI* NC≠non-VaMCI*	n.s.	NC≠VaMCI* NC≠non-VaMCI*
	Bella et al. 2011	-	NC≠VaMCI*	-	-	-	-	-	n.s.
	Kim et al., 2012	-	n.s.	VaMCI≠VaD**	VaMCI≠VaD**	VaMCI≠VaD**	VaMCI≠VaD**	VaMCI≠VaD**	VaMCI≠VaD**
	Lee et al., 2014	-	n.s.	n.s.	n.s.	n.s.	Non-VaMCI≠VaMCI**	n.s.	Non-VaMCI≠VaMCI*
	Sheorajpanday et al., 2014	n.s.	-	Non-VaMCI≠VaMCI**	Non-VaMCI≠VaMCI**	n.s.	n.s.	n.s.	n.s.
Moderate or severe WMH and/or <5 lacunes	Norlund et al., 2007	NC≠VaMCI** Non-VaMCI≠VaMCI*	NC≠VaMCI*	NC≠VaMCI** Non-VaMCI≠VaMCI*	NC≠VaMCI**	NC≠VaMCI* Non-VaMCI≠VaMCI*	NC≠VaMCI**	NC≠VaMCI** Non-VaMCI≠VaMCI*	NC≠VaMCI* NC≠non-VaMCI*
	Gainotti et al., 2008	-	NC≠VaMCI*	n.s.	n.s.	NC≠VaMCI*	NC≠VaMCI** Non-VaMCI≠VaMCI**	NC≠VaMCI** Non-VaMCI≠VaMCI**	NC≠VaMCI*
	Zhou et al., 2009a	-	NC≠VaMCI**	NC≠VaMCI**	NC≠VaMCI** Non-VaMCI≠VaMCI**	NC≠VaMCI**	NC≠VaMCI** Non-VaMCI≠VaMCI**	-	NC≠VaMCI**
	Zhou et al., 2009b	-	NC≠VaMCI**	NC≠VaMCI*	NC≠VaMCI**	NC≠VaMCI**	NC≠VaMCI**	-	NC≠VaMCI**
	Norlund et al., 2011	n.s.	n.s.	n.s.	n.s.	n.s.	Non-VaMCI≠VaMCI*	n.s.	n.s.
	Marra et al., 2011	-	n.s.	n.s.	n.s.	Non-VaMCI≠VaMCI**	Non-VaMCI≠VaMCI**	Non-VaMCI≠VaMCI**	n.s.
	Villeneuve et al., 2011	-	NC≠VaMCI*	-	NC≠VaMCI*	NC≠VaMCI*	NC≠VaMCI*	NC≠VaMCI*	NC≠VaMCI*
	Yi et al., 2012	-	-	-	-	-	NC≠VaMCI**	-	NC≠VaMCI**
	Sudo et al., 2013	NC≠VaMCI*	-	n.s.	n.s.	NC≠VaMCI*	n.s.	-	NC≠VaMCI*
	Brookes et al., 2015	CVD≠VaMCI**	-	CVD≠VaMCI**	-	-	CVD≠VaMCI**	-	CVD≠VaMCI**

* p<0.05;

** p<0.01

**Table 4:** Cognitive domains and corresponding neuropsychological tasks.

Cognitive functions		Tests
Executive Function (EF)	Shifting	Wisconsin Card Sorting Test (WMST): perseveration, Trail-Making Test (TMT) B, Dual task, Num­ber-Letter sequencing
	Inhibition	WCST: non-perseverative errors and categories, Go/No go, Fist/Edge/Palm sequence, Stroop test
	Working Memory/Update	Digit Span forward and backwards, Corsi test, Parallel Serial Mental Operations, CAMCOG: Work­ing Memory Subtest, Number and Letter sequencing.
	Less specific EF tests	Category and Letter verbal fluency, Luria loop, Raven matrices, Barcelona test (Abstraction sub­test), CAMCOG: Abstraction subtest, COWAT, Digit-Symbol substitution test, Cognitive estimation test, WAIS-III(picture interpretation and arrangement, Clock Drawing Test/CLOX 1 (spontaneous drawing), California Card Sorting Test
Visuospatial/visuoconstructive abilities		Block design, Rey figure: copy, TMT A, Visual Object and Space Perception, Lines cancellation test, Clock Drawing Test/CLOX 2. (copy), Multiple Features Target Cancellation
Memory		Prose recall, Babcock Story Recall test, Wechsler Memory Scale-Revised, Memory Assessment Scales, Hopkins Verbal Learning Test, Rey figure: recall and recognition, CAMCOG: Memory sub­test, Five-item memory test
Language		Token test, Boston Naming test, Assessment of Subtle Language Deficits
Global Cognition		MMSE, CAMCOG, BMET

MMSE: Mini-Mental State Examination; CAMCOG: Cambridge Cognitive Examination part of the Cambridge Examination for. Mental Disorders of the Elderly (CAMDEX).; BMET: Brief Memory and Executive Test; WAIS-III: Wechsler Adult Intelligence Scale, 3rd Edition.

Studies using the severe WMH and/or more than 5 lacunes criteria evidenced significant differences among VaMCI, VaD and controls in EF, Memory and Visuospatial/ Visuoconstructive tasks. Tests that measured "impure" and unspecific EF dimensions, labeled herein as "less specific EF tasks", consistently distinguished VaMCI from the other groups, while Working Memory Tasks appear to be less sensitive for detection of VaMCI. As expected, performances in Memory tests identified non-VaMCI from VaMCI, but also differentiated VaMCI from controls in some studies. Global cognitive measures were more accurate in distinguishing VaMCI from controls and VaD than from non-VaMCI.

When moderate-to-severe WMH and/or less than 5 lacunes were used as criteria for CVD, EF, Memory, Visuospatial abilities tests, as well as Global Cognitive assessment, differentiated VaMCI from controls in most studies. Memory and Language tests were accurate measures in distinguishing VaMCI from non-VaMCI. Among EF dimensions, Inhibition and unspecific EF tests consistently detected VaMCI from controls in the selected studies.

## DISCUSSION

The idea that VCI comprises a spectrum of different stages of vascular-related cognitive impairment may suggest that dementia can be preceded by subtle cognitive changes associated with CVD.^41^ However, the boundaries of vascular burden that mark the earliest clinical stages of CVD still need to be defined. The importance of establishing the milder pathological clinical phase of VCI resides in the fact that early identification of cognitive decline associated with CVD might allow adequate control of vascular risk factors, so as to prevent progression to dementia. In this perspective, the adoption of the neuroimaging criteria proposed by Erkinjuntti et al. for Binswanger Disease (2000) identified cases in which white-matter injury is already extensive, that may limit the effect-sizes of prophylactic actions. The present article reviewed data suggestive of expressive cognitive changes associated with moderate-to-severe WMH and less than 5 lacunes. Identification of those subjects might allow more effective actions in preventing progression of cognitive decline.

Studies using either severe or moderate-to-severe CVD criteria demonstrated that EF performances could distinguish VaMCI from non-VaMCI, VaD and normal controls. Global and "impure" EF tasks, comprising instruments that assess multiple and complex EF abilities, such as planning, reasoning, decision-making and abstract thinking, appear to be more sensitive in discriminating VaMCI from controls than specific and "pure" EF measures, even in the group with moderate WMH. Data from functional neuroimaging studies suggested that those "higher level" EF may recruit diverse areas in the prefrontal, parietal, medial and superior temporal cortices, and subcortical structures (amygdala, thalamus and cerebellum).^42,43^ These findings indicate that complex EF may result from the fine integration of many different cortical areas and subcortical regions, which depends on an extensive and delicate network of neural projections.^44^ Moderate white-matter changes, represented by periventricular smooth halo and beginning confluent deep WMH on neuroimaging, may be sufficient to interrupt segments of inter-cortical and/or cortical-subcortical loops, leading to disconnection of areas associated with complex EF.^45^

On the other hand, data on the accuracy of more specific EF measures in distinguishing controls, VaMCI and non-VaMCI appeared to be inconsistent, as observed in relation to shifting tasks. Performances in inhibition tasks were significantly worse in VaMCI subjects than in controls in most of the studies with moderate-to-severe CVD. This finding might suggest an early impairment of inhibitory control in VCI patients, which is in line with a previous prospective study.^46^ Interconnections among prefrontal cortex, subcortical regions and posterior areas might be interrupted in those patients, leading to loss of prefrontal inhibitory inputs over cortical-subcortical networks associated with task-irrelevant distracters.^47,48^ Among the severe CVD group, only two studies performed a similar analysis, showing conflicting results. Furthermore, working memory tasks were consistently inaccurate in differentiating VaMCI from non-VaMCI in most studies. Reports of impairments in working memory in amnestic MCI are abundant in the literature; thus, both Vascular and amnestic MCI might share, through different pathological mechanisms, similar prefrontal and cingulate dysfunction associated with working memory abilities.^49^

Non-executive cognitive domains were also tested in the studies. As expected, episodic memory tasks were more impaired in "atrophic" MCI than in VaMCI, in most of the studies. Yet, the finding that episodic memory performances were significantly poorer in VaMCI than in controls may highlight the role of the prefrontal cortex for the retrieval of information. Recent evidence suggested that left prefrontal cortex may participate in the recall process through the use of environmental cues and the ability to inhibit irrelevant memories during a task.^50^ Also, not surprisingly, impairments in visuospatial and visuoconstructive abilities were more prominent in VaMCI than in non-VaMCI and controls. Those alterations have been associated with CVD in different studies.^51,52^ Finally, screening tests (MMSE) and global cognitive assessment instruments (CAMCOG, BMET) identified VaMCI from controls in many studies and also from non-VaMCI in a smaller number of articles. Differently from longer neuropsychological batteries, many studies reported ceiling-effects for MMSE in samples comprising single-domain MCI subjects. However, evidence suggested that it may present similar accuracy in detecting multidomain impairments as compared with the Montreal Cognitive Assessment (MoCA) and the Addenbrooke's Cognitive Examination-Revised (ACE-R).^53^

Some other issues should be addressed. Despite slight variations, specially related to the instruments used to detect cognitive impairment and to the degree of deviation from normal cognition necessary to characterize the disorder, the clinical criteria proposed by Petersen et al. for MCI (2001) were adopted almost unchanged by most of the authors.^54^ This fact might indicate that, albeit past criticisms were directed to the disorder's construct validity, the use of the clinical entity described by Petersen et al. has largely prevailed among clinical studies.^55^ Conversely, other operational criteria have shown to be not optimal to identify MCI associated with CVD. Salvadori et al. (2015) reported that the criteria proposed by Winblad et al. (2004) might overlook non-amnestic MCI presentations.^52^

There are limitations in this review that need to be commented. Different terminologies used to describe periventricular and deep WMH and imprecise expressions (e.g., "patchy WMH", "diffuse WMH", "smooth halo" and "caps"), present in different criteria make it difficult to compare lesion loads across studies. Furthermore, the characterization of periventricular/deep WMH itself has been object of divergence by some authors, who adopted different distances between the ventricle's margin and the lesion to define it as "periventricular" or "deep".^56,57^ Moreover, tasks classified as assessing a specific aspect of EF may not be pure measures of that process, since they commonly require other EF and non-EF features. Models of EF as a unique or multiple constructs have been proposed and there is no agreement regarding neuropsychological tests that may thoroughly assess all of its aspects. Further studies using confirmatory factor-analysis of EF measures may allow the establishment of cognitive batteries comprising tests that evaluate complementary processes of EF.

The present review evidenced that the choice of neuroimaging criteria to characterize CVD in MCI subjects did not result in groups with different cognitive profiles. One possible hypothesis is the complex nature of subcortical disease, in which vascular and non-vascular (e.g., Alzheimer's disease, multiple sclerosis) events often interact, ultimately resulting in WM disconnection and cognitive impairment.^58,59^ In addition, as suggested by Pasi et al. (2015), that may also be due to the fact that cognitive tests may lose their accuracy in distinguishing groups of patients once certain degree of vascular lesions is reached.^60^

In conclusion, evidence in the literature suggested that the use of moderate-to-severe WMH and less than 5 lacunar infarcts as the earliest pathological neuroimaging presentation of CVD appear to be appropriate. Future operational criteria for VCI, especially for VaMCI, should place more emphasis in the clinical relevance of the early diagnosis. As mentioned, this measure may allow early intervention over risk-factors, with opportune effect in preventing progression to VaD.
